# [^68^Ga]Ga‐PSMA‐11 PET/CT for baseline staging of high‐risk prostate cancer: A real‐world study

**DOI:** 10.1002/bco2.70209

**Published:** 2026-06-23

**Authors:** Amy Rose Sharkey, Paul Bassett, Manil Subesinghe, Gurdip Azad, Ajay Aggarwal, Simon Hughes, Prokar Dasgupta, Rick Popert, Ben Challacombe, Gary J. R. Cook

**Affiliations:** ^1^ School of Biomedical Engineering and Imaging Sciences King's College London, St. Thomas' Hospital London UK; ^2^ Guy's and St. Thomas' NHS Foundation Trust London UK; ^3^ Statsconsultancy Ltd, Longwood Lane Amersham UK; ^4^ Faculty of Life Sciences and Medicine King's College London, King's Health Partners, King's College London UK; ^5^ London & Guy's and St. Thomas' PET Centre, St. Thomas' Hospital London UK

**Keywords:** Metastatic disease, Prostate cancer, PSMA‐11 PET/CT

## Abstract

**Objective:**

To use real‐world data to determine the clinical risk factors that are predictive of metastatic disease in high‐risk prostate cancer (PCa) patients undergoing staging [^68^Ga]Ga‐Prostate‐Specific Membrane Antigen (PSMA)‐11 positron emission tomography combined with computed tomography (PET/CT).

**Methods:**

Subjects with newly diagnosed PCa who underwent [^68^Ga]Ga‐PSMA‐11 PET/CT between 1/2/20 and 1/4/23, with one or more of three major risk factors (prostate‐specific antigen (PSA) ≥ 20, MRI T‐stage ≥ 3 or International Society of Urological Pathology (ISUP) grade ≥ 3) were included. Metrics collected included [^68^Ga]Ga‐PSMA‐11 PET/CT primary index tumour maximum standardized uptake value (SUV_max_), TNM stage, tumour histology, patient age, body mass index and treatment type.

**Results:**

A total of 525 subjects were eligible for inclusion. A total of 22.1% had nodal or distant metastases on the baseline [^68^Ga]Ga‐PSMA‐11 PET/CT (11.8% with one major risk factor, 25.6% with two and 43.5% with three). All three major risk factors (PSA ≥ 20, MRI T‐stage ≥ 3, ISUP grade ≥ 3) and the presence of a higher percentage of positive biopsy cores were significant independent risk factors for the presence of metastatic disease on multivariable analysis. Primary index tumour SUV_max_ was associated with clinical risk factors, including ISUP grade in the surgical cohort. A total of 146 (27.8%) subjects underwent a radical prostatectomy, and 379 (72.2%) received non‐surgical management (including 242 (46.1%) who received curative intent radiotherapy (RT)).

**Conclusion:**

This study provides real‐world validation of the clinical risk factors used for the ProPSMA study for [^68^Ga]Ga‐PSMA‐11 PET/CT scan eligibility, which represent significant independent risk factors for the presence of nodal or distant metastases on baseline [^68^Ga]Ga‐PSMA‐11 PET/CT.

## 1 INTRODUCTION

Prostate cancer (PCa) is one of the most common cancers in men worldwide, with estimates suggesting the number of new cases annually will rise from 1.4 million in 2020 to 2.9 million by 2040.^1^ Screening for PCa is commonly via the serum tumour biomarker prostate‐specific antigen (PSA); if elevated, multiparametric magnetic resonance imaging (mpMRI) is performed to assess for the presence of primary PCa and to guide biopsies. Multiple risk factors are associated with poor outcomes in PCa, including increased age, higher PSA, higher grade histology, MRI tumour stage (T‐stage) and the presence of metastases. Together, these parameters are incorporated into a strategy of PCa staging that predicts prognosis and helps to guide treatment.^2^

One of the most significant prognostic factors in PCa is the presence of metastatic disease at baseline, defined here as the presence of any nodal or distant metastatic disease. Assessment of the spread of PCa is via imaging, traditionally with computed tomography (CT) and bone scintigraphy, now increasingly replaced by prostate‐specific membrane antigen (PSMA) PET/CT. PSMA is a cell‐surface glycoprotein overexpressed on PCa cells, and radiolabelled small molecules can bind with affinity to PSMA, enabling whole‐body tumour‐specific imaging with PET/CT. This type of imaging can facilitate the early diagnosis of both local spread to lymph nodes and distant metastatic disease. The prospective, randomized, multicentre ProPSMA trial, including men diagnosed with localized high‐risk PCa, revealed that [^68^Ga]Ga‐PSMA‐11 PET/CT offered a 27% (95% CI 23–31) greater diagnostic accuracy than conventional imaging, with an accuracy of 92% for detecting pelvic nodal or distant metastases (reference standard was a predefined composite panel encompassing histopathologic, imaging, clinical and biochemical findings), compared with 65% for standard imaging comprising the combination of CT and bone scans using single photon emission computed tomography combined with CT (SPECT/CT).^3^

The improved detection of metastases with [^68^Ga]Ga‐PSMA‐11 PET/CT is key in treatment planning; identification of metastatic spread at baseline allows appropriate treatment options to be offered, with more radical treatment options available for those without metastatic disease. [^68^Ga]Ga‐PSMA‐11 PET/CT has been shown to drive management changes in 28% of men, compared with 15% for conventional imaging.^3^ Furthermore, the maximum standardized uptake value (SUV_max_) of the primary prostatic tumour at diagnosis, as measured by PSMA PET, has been associated with oncological outcomes, with focal PSMA ligand uptake (SUV_max_ > 6) thought to enable detection of clinically significant tumours.^4^ A recent meta‐analysis revealed preoperative intraprostatic PSMA SUV_max_ increases with higher ISUP grade and pathological tumour stage, and that higher SUV_max_ is associated with reduced biochemical recurrence (BCR) free survival.^5^

In our tertiary institution, the presence of nodal or distant metastases alters management, with those without metastases offered radical prostatectomy, radiotherapy or brachytherapy, and those with metastases offered alternative treatment strategies. Subjects with one or more of three major risk factors (PSA ≥ 20 ng/ml, MRI T‐stage ≥ 3, ISUP grade ≥ 3) undergo a [^68^Ga]Ga‐PSMA‐11 PET/CT as a primary staging scan to assess for metastases, in line with the risk factors featured in the ProPSMA study.^3^ We aim to retrospectively analyse the data from our large real‐world cohort to assess which risk factors are significant for the presence of baseline metastatic disease.

## 2 MATERIALS AND METHODS

### 2.1 Patient population

Institutional approval was acquired for this single tertiary referral centre retrospective study. Consecutive treatment‐naïve subjects with newly diagnosed PCa who underwent a baseline staging [^68^Ga]Ga‐PSMA‐11 PET/CT study between 1/2/20–1/4/23, with at least one major risk factor (PSA ≥ 20, T‐stage ≥ 3 or ISUP grade ≥ 3) were included for analysis. Exclusion criteria comprised those with previous or recurrent PCa at the time of the [^68^Ga]Ga‐PSMA‐11 PET/CT, those who underwent a [^68^Ga]Ga‐PSMA‐11 PET/CT with no baseline clinical risk factors, and those subjects for whom histology was not available for review.

### 2.2 PSMA PET/CT protocol and image evaluation

Subjects were scanned on either a Siemens Biograph mCT (Erlangen, Germany) or a GE Discovery 710 (Waukesha, US) PET/CT scanner. No specific patient preparation was required except bladder voiding immediately before imaging. All patients were injected intravenously with [^68^Ga]Ga‐PSMA‐11 (mean, 169.6 ± 16.5 MBq). At 60 minutes, a scan was acquired from pelvis to skull base at four minutes per bed position with an axial field of view of 15.7 cm and an 11‐slice overlap between bed positions. A low‐dose CT scan (140 kV; mAs, 15–100; noise index, 40; rotation time, 0.5 s; and collimation, 40 mm) was obtained at the start of imaging to provide attenuation correction and an anatomic reference. PET scans were reconstructed using time‐of‐flight (ToF) data (Bayesian penalized‐likelihood reconstruction algorithm (Q.CLEAR); GE Healthcare: point‐spread function modelling (True TOF); Siemens).

Image evaluation was performed using Hermes Medical Solutions (Stockholm, Sweden) Gold software. Scans are routinely evaluated and prospectively interpreted according to the PROMISE criteria^11^ at our institution. The determination of the maximum standardized uptake values (SUV_max_) in the primary prostatic tumour was performed via a semi‐automated region of interest (ROI). All scans were evaluated by one of four consultant nuclear medicine physicians/radionuclide radiologists for activity in the prostate and TNM status and reanalysed by one of these doctors (GC) before tumour board discussion.

### 2.3 Metrics and statistical analysis

Metrics collected included age, weight, height, MRI T‐stage, [^68^Ga]Ga‐PSMA‐11 PET/CT results (TNM stage, primary prostatic lesion SUV_max_), histology results (Gleason score, ISUP grade, number of prostate sites biopsied, percentage of positive cores, presence of cribriform pattern, perineural invasion, maximum tumour length), baseline and current PSA, treatment type and follow‐up period

Anonymized data were collected on Microsoft Excel and statistically analysed using GraphPad Prism. Univariable and multivariable binary logistic regression models were calculated. The dependent variable in all cases was: metastasis (lymph node, visceral or skeletal) occurred (yes or no). The univariable model included age, body mass index (BMI), MRI T‐stage, the most recent PSA prior to the [^68^Ga]Ga‐PSMA‐11 PET/CT scan, ISUP grade, SUV_max_ of the primary prostate tumour and other histological parameters (% positive tumour sites at biopsy, % positive prostatic cores, presence of perineural invasion, presence of cribriform pattern, maximum tumour length). For the multivariable analysis, variance inflation factors were calculated to assess collinearity between predictor variables. A backwards selection procedure was used to choose the variables in the final model. A p value of <0.05 was considered significant. The relationship between the SUV_max_ of the primary prostate lesion with ISUP grade and other risk factors was also evaluated. A subanalysis was performed to assess baseline metastatic disease in subjects with intermediate unfavourable disease (defined as a single risk factor of Gleason 4 + 3 disease).

## 3 RESULTS

**FIGURE 1:**
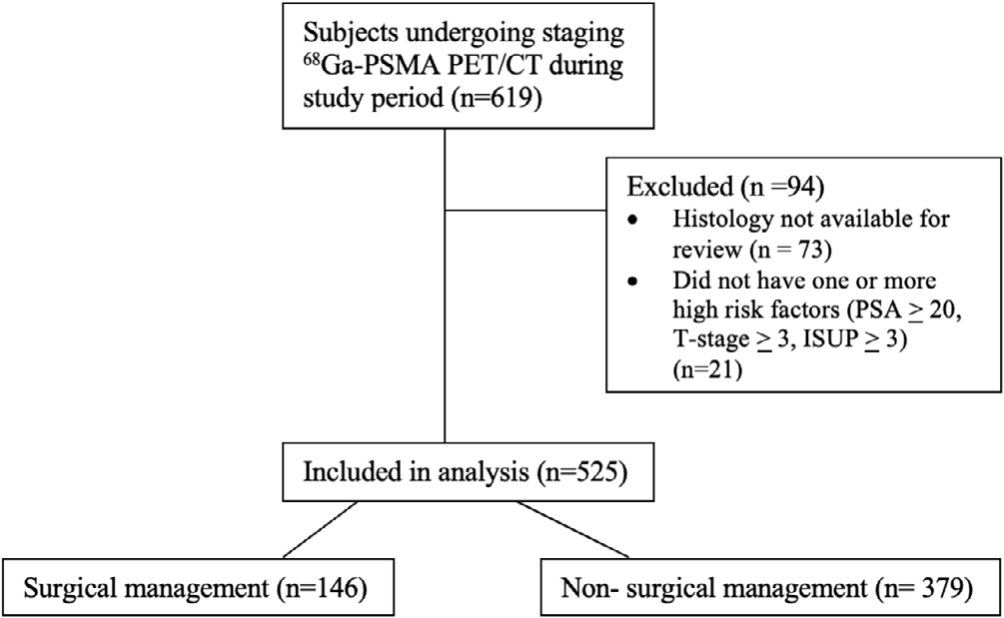
CONSORT flowchart.

619 subjects underwent a [^68^Ga]Ga‐PSMA‐11 PET/CT during the study period; 73 were excluded as their histology was not available for review (performed elsewhere and not retrievable), and a further 21 were excluded as they did not have one of the risk factors (PSA ≥ 20, MRI T‐stage ≥ 3, ISUP grade ≥ 3) required to meet our institutional criteria to be eligible for a primary staging [^68^Ga]Ga‐PSMA‐11 PET/CT study (subject inclusion/exclusion flow chart is demonstrated in Figure 1.) 525 subjects were eligible for inclusion. The mean subject age was 67 ± 8 years, with a mean BMI of 27.6 ± 4.8 and mean PSA of 23.0 ± 32.7 ng/ml at diagnosis.

22.1% (116/525) of subjects had metastatic disease at the primary staging [^68^Ga]Ga‐PSMA‐11 PET/CT scan. 19.6% (103/525) had N1 stage disease (PSMA PET positive pelvic nodes below common iliac nodes), 7.3% (38/525) had M1a stage disease, 7.6% (40/525) had M1b stage disease and 0.6% (3/525) had M1c stage disease. The risk of metastatic disease increased with the number of major risk factors (PSA ≥ 20 ng/ml, MRI T‐stage ≥ 3, ISUP grade ≥ 3) present: 11.8% for a single risk factor, 25.6% for any two risk factors and 43.5% if all three risk factors were present.

There were 117 subjects with unfavourable intermediate risk disease: 45 subjects were managed surgically, of whom none had had metastatic disease at diagnosis, and 72 were managed non‐surgically, of whom nine had metastases at diagnosis (six subjects with N1 disease and three with M1b disease). Overall, 9/117 (7.7%) of unfavourable intermediate risk based on Gleason score alone had metastatic disease at the baseline staging scan.

### 3.1 Univariable analysis

Univariable analysis indicated that PSA, ISUP grade and MRI T‐stage were all significantly associated with metastatic disease. In terms of histopathology, the percentage of positive cores and positive sites, and perineurial invasion, as well as SUV_max_, were all significantly associated with metastatic disease (Table 1). There was also slight evidence that age was associated with the outcome, but this result did not reach statistical significance (p = 0.09). The results were not found to be significant for either cribriform pattern (p = 0.20) or BMI (p = 0.34).

**TABLE 1:** Significant univariable associations with metastatic disease.

Variable	Category	Metastases n/N (%)	Odds Ratio (95% CI)	*p*‐value
PSA (baseline)	< 20	59/360 (16%)	1	**<0.001**
	≥ 20	58/182 (32%)	2.39 (1.57, 3.63)	
ISUP grade	< 3	8/84 (10%)	1	**0.006**
	≥ 3	106/456 (23%)	2.88 (1.35, 6.15)	
MRI T‐stage	T1/T2	35/286 (12%)	1	**<0.001**
	T3/T4	79/251 (31%)	3.29 (2.11, 5.13)	
% positive cores**	‐	‐	1.27 (1.16, 1.38)	**<0.001**
% positive sites**	‐	‐	1.33 (1.19, 1.49)	**<0.001**
Perineurial invasion	No	38/259 (15%)	1	**0.007**
	Yes	60/247 (24%)	1.87 (1.19, 2.93)	
SUV_max_ **	‐	‐	1.16 (1.02, 1.32)	**0.02**

* Odds ratio given for a 5‐unit increase in variable.

** Odds ratio given for a 10‐unit increase in variable.

### 3.2 Multivariable analysis

No notable collinearity between predictor variables was observed. Multivariable analysis indicated that PSA, ISUP grade, MRI T‐stage and the percentage of positive prostate biopsy sites were all significantly associated with metastatic disease (Table 2). Patients with MRI T‐stage ≥ 3 had odds of metastatic disease that were twice those of patients with a lower stage. Those with ISUP grade ≥ 3 had 3.9 times the odds of metastatic disease compared to patients with a lower stage, whilst a higher PSA value (≥20) was associated with a 1.8‐fold increase in the odds of metastases. Every 10% increase in the percentage of positive cores at biopsy was associated with a 24% increase in the odds of metastatic disease.

**TABLE 2:** Significant Multivariable associations with metastatic disease.

Variable	Category	Odds Ratio (95% CI)	*p*‐value
PSA (original)	< 20	1	**0.03**
	≥ 20	1.79 (1.07, 3.00)	
ISUP grade	< 3	1	**0.003**
	≥ 3	3.89 (1.59, 9.51)	
MRI T‐stage	T1/T2	1	**0.004**
	T3/T4	2.15 (1.28, 3.62)	
% positive cores at biopsy**	‐	1.24 (1.10, 1.41)	**0.001**

** Odds ratio given for a 10‐unit increase in variable.

### 3.3 Primary prostatic lesion SUV_max_

**FIGURE 2:**
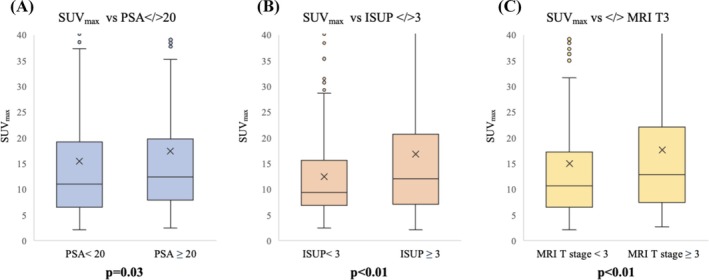
Relationship of SUV_max_ of the primary prostatic lesion with PSA, ISUP grade and MRI T stage. **A**, **B** and **C** illustrate the primary prostatic lesion SUV_max_ vs. PSA, ISUP grade and MRI T‐stage, respectively.

**FIGURE 3:**
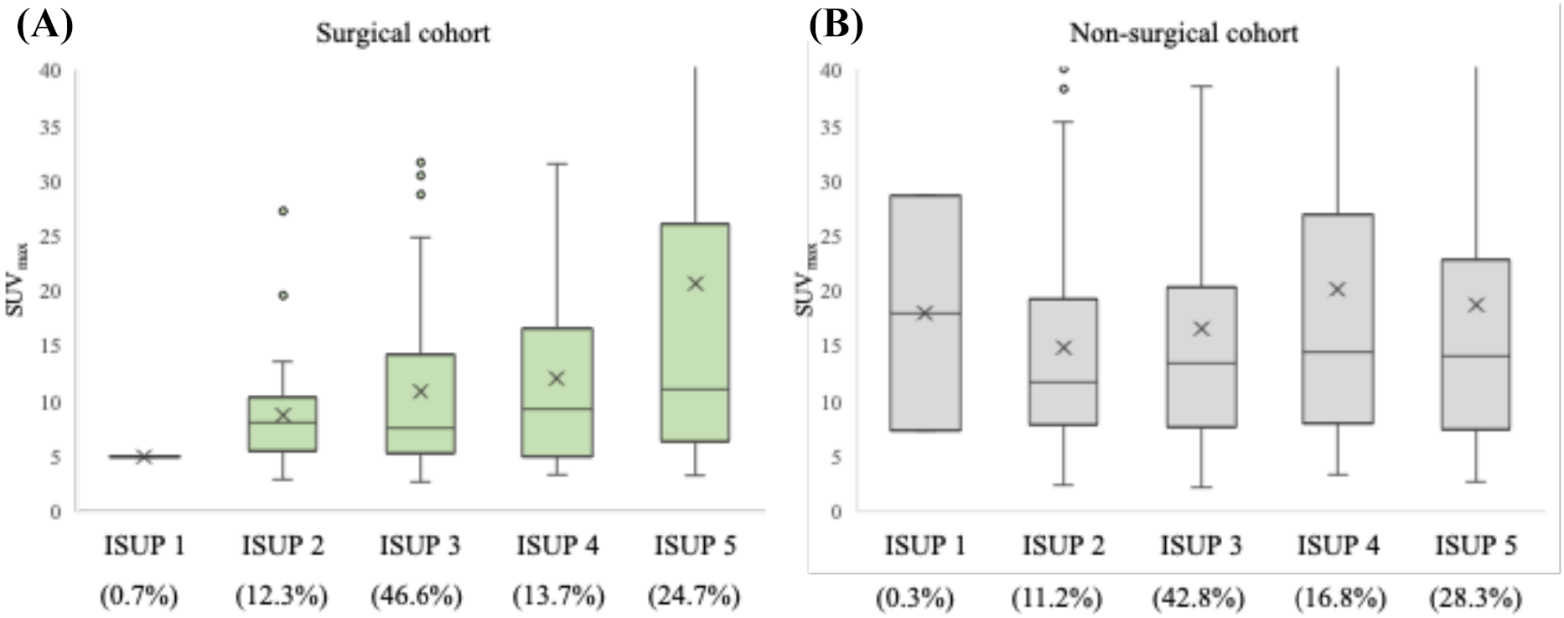
**A** illustrates ISUP grading in the surgical cohort, and **B** illustrates ISUP grading in the non‐ surgical cohort.

Although the primary prostatic lesion SUV_max_ was associated with risk of metastases on univariable analysis (p = 0.02), it was not found to be a significant independent predictor of metastatic disease on multivariable analysis. In the surgical cohort, where there was definitive prostatectomy histology, although there was high variability in the relationship between the primary lesion SUV_max_ within individual ISUP grades, there was a trend for higher SUV_max_ in those with higher ISUP grade and higher SUV_max_ when PSA ≥ 20 and T‐stage ≥ 3 (Figure 2). The trend for increasing SUV_max_ with increasing ISUP grade was not seen in the non‐surgical cohort (Figure 3).

### 3.4 Treatment type

In our cohort, 146 subjects received surgical management (RP) and 379 received non‐surgical management. Non‐surgical treatment options included radiotherapy, brachytherapy and hormone treatment (Table 3). We cannot accurately assess biochemical recurrence in this study due to the short follow‐up period.

**TABLE 3:** Management of subjects.

Management option		Number of subjects	Follow‐up time (median days ± range)
Curative treatment	Radical prostatectomy	146	382 (242–1035)
	RT plus hormones	242	372 (14–1385)
	Brachytherapy	11	173 (55–562)
	Cryotherapy	2	78 (24–131)
Non‐curative treatment	Hormones alone	93	
	Active surveillance, watchful waiting or declined treatment	15	
	Unknown	13	

## 4 DISCUSSION

This analysis of our large tertiary cohort of newly diagnosed PCa patients demonstrates that PSA ≥ 20, MRI T stage ≥3 or ISUP grade ≥3, as well as a higher percentage of positive prostate biopsy cores, are all independently and significantly associated with the presence of metastatic disease at the primary staging [^68^Ga]Ga‐PSMA‐11 PET/CT. The risk of metastases was 11.8% with a single risk factor, 25.6% for any of two risk factors and 43.5% if all three risk factors (PSA ≥ 20, MRI T‐stage ≥3 or ISUP grade ≥3) that were used in the ProPSMA trial^3^ were present. This study provides data from a large real world cohort to validate the proPSMA findings, and entry eligibility risk factors the ProPSMA trial adopted.

### 4.1 Assessing risk of metastatic spread

The accurate assessment of metastatic PCa spread is important; while 5‐year survival rates are excellent for localized PCa, lifespan is limited for patients with a distant tumour burden. Siegel et al.^6^ reported a 5‐year relative survival rate of 31% in men with metastatic disease. For those patients with metastatic PCa, treatment intensification strategies can improve survival. These management strategies are often altered on the basis of PSMA PET/CT,^3^ so accurate stratification of which patients receive a primary PSMA PET/CT is vital. The utility of PSMA PET/CT is now well established in high‐risk disease,^7^ whereas patient selection in intermediate‐risk disease remains less certain.^8^ We report 117 subjects with unfavourable intermediate‐risk disease; of these, 7.7% had metastases at baseline staging, suggesting there is merit in this cohort of patients undergoing baseline PSMA PET imaging. These findings are in line with others, such as those of Yaxley et al.,^8^ who report a 5.2% risk of metastases at baseline in their unfavourable intermediate risk group vs 19.9% in those with high risk PCa. Similarly, Hagens et al.^9^ found that in their population of 396 men with newly diagnosed unfavourable intermediate‐risk PCa, 29 (7.3%) had metastases, and a tumour stage of at least T3 on MRI and more than 50% positive prostate biopsies were found to be independently associated with metastatic disease on PSMA PET/CT.

There are several studies assessing prognostic factors in prostate cancer,^9^, ^10^ and several predictive models have been developed to assess prognosis in PCa, primarily using clinical, biochemical and pathological data points. Muehlematter et al.^12^ developed a multivariable [^68^Ga]Ga‐PSMA‐11 PET‐based prediction model for the assessment of lymph node involvement in men with intermediate or high‐risk PCa, combining PSA, Gleason grade, [^68^Ga]Ga‐PSMA‐11 positive volume of the primary tumour and PSMA nodal status, finding PSMA PET/CT can improve lymph node involvement prediction in intermediate to high‐risk PCa patients undergoing primary staging, especially when combined with clinical parameters.

The inputs to these predictive models are of critical importance. Allowing for existing reports predominantly aimed at determining sensitivity and specificity (including a recent meta‐analysis^13^) or correlating SUV with Gleason score or investigating in a biochemical recurrence setting, there are surprisingly few comparable data in the literature^14^, ^15^, ^16^ to support the proPSMA proposed risk factors when staging prostate cancer at diagnosis outside of a formal trial environment and in a real‐world situation. The proPSMA study did not perform a multivariable analysis to evaluate individual risk factors. Here, we provide validating real‐world data for the risk factors used in the proPSMA trial, believing that it is important that supportive data from a real‐world setting outside a formal prospective trial are reported.

### 4.2 Role of SUV_max_

The SUV_max_ of the primary prostatic tumour has been associated with oncological outcomes.^4^ We report that in univariable analysis, a higher SUV_max_ of the primary prostatic lesion was associated with the presence of metastatic disease, but this was not reproduced on multivariable analysis, possibly due to factors such as other studies using different PSMA ligands and scanning protocols. A previous real‐world study involving a smaller Australian cohort found that SUV_max_ of the primary prostate tumour was associated with the extent of metastatic disease.^6^ Koerber et al.^17^ evaluated SUV_max_ and the risk of metastatic disease (without histopathological correlation), finding a statistically significant higher SUV_max_ in men with metastatic disease than without distant metastases (median SUV_max_ 16.1 vs. 11.2; *p* < 0.001). However, that study included PSMA PET/CT studies using both [^18^F]PSMA‐1007 and [^68^Ga]Ga‐PSMA‐11, which may have affected their SUV_max_ results and may explain why we did not find such a correlation in our larger cohort using only [^68^Ga]Ga‐PSMA‐11.

We report that higher SUV_max_ measurements were associated with PSA ≥ 20 and MRI T‐stage ≥ 3. The primary prostatic lesion SUV_max_ varied widely for each ISUP grade (Figure 2) but there was a trend for higher SUV_max_ in higher ISUP grade lesions in the surgical cohort. This is in keeping with previous literature, finding SUV_max_ of intraprostatic, malignant lesions to be highly correlated with several clinical parameters, such as the Gleason Score and PSA.^18^ Lack of a relationship between SUV_max_ and ISUP grade in the non‐surgical cohort is unexplained but may have been partly due to the lower reliability of ISUP grading on biopsy rather than prostatectomy.

Another factor to consider is that there is known variability between institutions in SUV_max_ measurements, due to scanner factors and reconstruction parameters. Interestingly, some models have chosen a cut‐off point of SUV_max_ to make it into a dichotomous categorical variable rather than a continuous variable, which could go some way to addressing this variability; although the cut off is difficult to choose, with one previous study suggesting a cut‐off SUV_max_ value of 11.9.^17^ One more recent approach is the development of a prognostic risk score for PCa based on PSMA PET‐derived organ‐specific tumour volumes,^19^ which showed a good model fit for predicting overall survival, although the study included a mixture of patients at different stages in the natural history of the disease. Similarly, Siefert et al.,^19^ in a retrospective study including all groups of prostate cancer patients, have evaluated more complex parameters, including PSMA PET volumetric and radiomic predictors, which show promise but are not of current practical use in a clinical setting.

### 4.3 Limitations

A limitation of the study is the retrospective single‐centre design with potential for referral bias, although cases were only included where local guidelines for PSMA referral had been adhered to. There are some factors of interest which are not included in routine data collection at our centre, which would be of analytic interest, such as subject race or subject family history of PCa, which limits our analysis. Further bias may be introduced by scanner heterogeneity. Our follow‐up time is too short to make a valid assessment of BCR, with a median of approximately one year in both cohorts.

## 5 CONCLUSION

This large cohort analysis of subjects with a newly diagnosed high‐risk PCa provides confirmatory evidence that PSA *≥* 20, ISUP grade *≥* 3, MRI T‐stage *≥* 3, as well as the percentage of positive cores at prostatic biopsy, are significant independent predictors of the presence of metastases on [^68^Ga]Ga‐PSMA‐11 PET/CT, providing real‐world validation of the results of the ProPSMA trial and entry eligibility risk factors. Primary prostatic lesion SUV_max_ was associated with clinical risk factors, including ISUP grade in the surgical cohort, but did not represent an independent risk factor for the presence of metastatic disease.

## AUTHOR CONTRIBUTIONS

Gary John Russell Cook proposed the idea of the manuscript. Amy Rose Sharkey performed data collection and analysis and prepared the manuscript draft. Paul Bassett assisted with statistical analysis. All other authors reviewed and provided clinical input into the manuscript.

## CONFLICT OF INTEREST STATEMENT

Prokar Dasgupta recognizes support from the Wellcome Trust for Surgical and Interventional Engineering, the London Institute for Healthcare Engineering (LIHE), the Hinduja‐King's Academy, Alberto Recordati, the King's‐Vattikuti Institute, The Urology Foundation and King's College London (KCL). No other potential conflicts of interest relevant to this article exist.
